# Supplementary material for: Patterns of Intron Gain and Loss in Fungi
Source: PLoS Biol. 2004 Nov 30;2(12):e422. doi: 10.1371/journal.pbio.0020422 (PMC532390; doi:10.1371/journal.pbio.0020422)
Supplement: Table S1 — Also available at http://genes.mit.edu/NielsenEtAl/. (4.3 MB ZIP). [file pbio.0020422.st001.zip › NielsenEtAl/html/76.html]

AN1403.1.NCU01013.1.MG10655.1.FG10080.1


```
 CLUSTAL W (1.82) Multiple Sequence Alignments - Introns Inserted


Sequence 1: MG10655.1	389 aa
Sequence 2: FG10080.1	386 aa
Sequence 3: NCU01013.1	385 aa
Sequence 4: AN1403.1	373 aa
Alignment Length: 389 aa
Number Identitical Residues: 266 aa
Alignment Score (without introns) 11812


MG10655.1 	MSFSSLVQDLSLRDANGARRRGLPRGDSSAPTIDDGASRTSHISRAMSYASTAATSVSIS
NCU01013.1	MSFSSLVQDLSLRDPNAPRR----RGDASVSTIDDRASRTSHISRAMSYASTAATSVSIS
FG10080.1 	MSFSSLVQDLSLRDANGARRPQISGPRSSASTLDD---RASHISRAMSYASTAATSVSIS
AN1403.1  	MSFSNLVSDLALRDSYEDRNSQISNARSQVT--------------ARSYTSTAATSVSIA
          	****.**.**:***.   *.   .   :...              * **:*********:

MG10655.1 	GDISSQLHGGYSHPLARSWQAERQLTK0SMLIYPLFISDSDDEEVLIPSLPDQYRRGINK
NCU01013.1	GDISSQLHGGYAHPLARSWQAERQLTK0SMLIYPLFVSDNDDEEVLIPSLPNQYRRGINR
FG10080.1 	GDISSQLHGGYFHPLARSWQAERQLTK0SMLIYPLFVTDGEGDMILVPSLPGQHQLSCDK
AN1403.1  	GDISSQLHSGYSHPLSRSWQAERQLTK0EMLIYPLFITDNPDEETPIPSLPNQHRRGLNR
          	********.** ***:*********** .*******::*. .:   :****.*:: . ::

MG10655.1 	VIPFLETLVHKGLRSVILFGVPMRPGTKDALGTSADDPQGPVMRSIRAIRQRFPQLFVVC
NCU01013.1	LVPFLEPLVHKGLRSVILFGVPLKPGTKDALGTAADDPEGPVIRSIRLLRQRFPQLYITV
FG10080.1 	LIPFLEPLVHKGLRSVMLFGVPMQAGTKDALGTAADDPEGPVIRAIQIIRRRFPQLFICC
AN1403.1  	LVPFLRPLVQKGLRSVILFGVPLHPSAKDALGTSADDPAGPVVQAIRLLRSRFPELYIVT
          	::***..**:******:*****::..:******:**** ***:::*: :* ***:*::  

MG10655.1 	DVCLCEYTSHGHCGILRDDGSLNNQLSVDRISDVAVAYARAGAHCVAPSDMNDGRIRAIK
NCU01013.1	DVCLCEYTSHGHCGILRDDGSLNNQLSVDRISDVALAYAKAGAHCVAPSDMNDGRIRAIK
FG10080.1 	DVCLCEYTSHGHCGILRDDGSLNNQLSVDRISDVAIAYAKAGAQCVAPSDMNDGRIRAIK
AN1403.1  	DVCICEYTSHGHCGILREDGTLDNAQSVDRISDVALAYASAGAHCVAPSDMNDGRVRAIK
          	***:*************:**:*:*  *********:*** ***:***********:****

MG10655.1 	LKLIEEGIAHNTLLMSYAAKFSGCLYGPFRDAAGSAPSFGDRKCYQLPHGGRGLARRAII
NCU01013.1	LKLIEEGIVHRVTLMSYAAKFSGCLYGPFRDAAGSCPSFGDRKCYQLPPGGRGLARRAIV
FG10080.1 	LKLIEEGIAHKTVLMSYSAKFSGCLYGPFRDAAGSAPSFGDRKCYQLPPGGRGLARRAIV
AN1403.1  	LKLIEAGLAHRVLLMSYSAKFSGCLYGPFRDAAGSCPSFGDRRCYQLPPGGRGLARRAIQ
          	***** *:.*.. ****:*****************.******:***** ********** 

MG10655.1 	RDINEGADVIMVKPASQYLDIISDAKDLGKDLPIAAYQVSGEYAMIHAAAKAGVFDLKTM
NCU01013.1	RDINEGADIIMVKPASQYLDIISDAKELGKDLPVAAYQVSGEFAMIHAAAKAGVFDLKAM
FG10080.1 	RDINEGADIIMVKPASQYLDIISDAKDLGKDLPVAAYQVSGEYAMIHAGAKAGVFDLKAM
AN1403.1  	RDIGEGADIIMVKPASSYLDIIRDAKELAKDIPIAAYQVSGEYAMIHAGAKAGVFDLKSM
          	***.****:*******.***** ***:*.**:*:********:*****.*********:*

MG10655.1 	AFESTEGILRAGATIVVSYFTPEFLDWLDS
NCU01013.1	AFEATEGILRAGATIIISYFVPEFLDWLST
FG10080.1 	AFESTEGILRAGATIVVSYFTPDFLDWLEN
AN1403.1  	AFESTEGILRAGAGIIVSYFVPEFLDWL--
          	***:********* *::***.*:*****
```
